# Supplementary material for: Assessing Differences in Attitudes toward Occupational Safety and Health Measures for Infection Control between Office and Assembly Line Employees during the COVID-19 Pandemic in Germany: A Cross-Sectional Analysis of Baseline Data from a Repeated Employee Survey
Source: Int J Environ Res Public Health. 2022 Dec 29;20(1):614. doi: 10.3390/ijerph20010614 (PMC9819385; doi:10.3390/ijerph20010614)

## SUPPLEMENTARY MATERIAL

The ***study protocol*** can be downloaded here: https://bmjopen.bmj.com/content/10/11/e043908 (Doi: 10.1136/bmjopen-2020-043908) (accessed on 25 August 2022) .

***Supplementary File S1:*** STROBE-checklist for cross-sectional studies
***Supplementary File S2:*** Employee survey components
***Supplementary File S3:*** Sensitivity analysis: results excluding observations with missing values

***Supplementary File S4:*** Attitude toward organisational, respectively personal, occupational infection control measures
***Supplementary File S5:*** CONSORT flow-chart

*Supplementary File S1:* **STROBE 2007 (v4) Statement—Checklist of items that should be included in reports of *cross-sectional studies***

| **Section/Topic** | **Item #** | **Recommendation** | **Reported on page #** |
|---|---|---|---|
| **Title and abstract** | 1 | (*a*) Indicate the study's design with a commonly used term in the title or the abstract | 1 |
| | | (*b*) Provide in the abstract an informative and balanced summary of what was done and what was found | 1 |
| **Introduction** | | | |
| Background/rationale | 2 | Explain the scientific background and rationale for the investigation being reported | 1-3 |
| Objectives | 3 | State specific objectives, including any prespecified hypotheses | 3 |
| **Methods** | | | |
| Study design | 4 | Present key elements of study design early in the paper | 3 |
| Setting | 5 | Describe the setting, locations, and relevant dates, including periods of recruitment, exposure, follow-up, and data collection | 3 |
| Participants | 6 | (*a*) Give the eligibility criteria, and the sources and methods of selection of participants | 3-4 |
| Variables | 7 | Clearly define all outcomes, exposures, predictors, potential confounders, and effect modifiers. Give diagnostic criteria, if applicable | 4-5 |
| Data sources/ measurement | 8* | For each variable of interest, give sources of data and details of methods of assessment (measurement). Describe comparability of assessment methods if there is more than one group | 4-5 |
| Bias | 9 | Describe any efforts to address potential sources of bias | 5-6 |
| Study size | 10 | Explain how the study size was arrived at | 6 & Supplementary File 5 |
| Quantitative variables | 11 | Explain how quantitative variables were handled in the analyses. If applicable, describe which groupings were chosen and why | 5-6 |
| Statistical methods | 12 | (*a*) Describe all statistical methods, including those used to control for confounding | 5-6 |
| | | (*b*) Describe any methods used to examine subgroups and interactions | 5-6 |
| | | (*c*) Explain how missing data were addressed | 5-6 |
| | | ~~(*d*) If applicable, describe analytical methods taking account of sampling strategy~~ | |
| | | (*e*) Describe any sensitivity analyses | 5-6; Supplementary File 5 |

| | | | |
|---|---|---|---|
| **Results** | | | |
| Participants | 13* | (a) Report numbers of individuals at each stage of study—e.g. numbers potentially eligible, examined for eligibility, confirmed eligible, included in the study, completing follow-up, and analysed | 6-7 & Supplementary File 5 |
| | | (b) Give reasons for non-participation at each stage | 14 & Supplementary File 5 |
| | | (c) Consider use of a flow diagram | 6 & Supplementary File 5 |
| Descriptive data | 14* | (a) Give characteristics of study participants (e.g., demographic, clinical, social) and information on exposures and potential confounders | 6-7 |
| | | (b) Indicate number of participants with missing data for each variable of interest | 6-7 |
| Outcome data | 15* | Report numbers of outcome events or summary measures | 6-7 |
| Main results | 16 | (*a*) Give unadjusted estimates and, if applicable, confounder-adjusted estimates and their precision (e.g., 95% confidence interval). Make clear which confounders were adjusted for and why they were included | 6-7 |
| | | (*b*) Report category boundaries when continuous variables were categorized | 6-7 |
| | | ~~(*c*) If relevant, consider translating estimates of relative risk into absolute risk for a meaningful time period~~ | |
| Other analyses | 17 | Report other analyses done—e.g., analyses of subgroups and interactions, and sensitivity analyses | Supplementary Files 3 and 4 |
| **Discussion** | | | |
| Key results | 18 | Summarise key results with reference to study objectives | 10 |
| Limitations | 19 | Discuss limitations of the study, taking into account sources of potential bias or imprecision. Discuss both direction and magnitude of any potential bias | 13 |
| Interpretation | 20 | Give a cautious overall interpretation of results considering objectives, limitations, multiplicity of analyses, results from similar studies, and other relevant evidence | 14 |
| Generalisability | 21 | Discuss the generalisability (external validity) of the study results | 13-14 |
| **Other information** | | | |
| Funding | 22 | Give the source of funding and the role of the funders for the present study and, if applicable, for the original study on which the present article is based | 14 |

~~*Give information separately for cases and controls in case-control studies and, if applicable, for exposed and unexposed groups in cohort and cross-sectional studies.~~

**Note:** An Explanation and Elaboration article discusses each checklist item and gives methodological background and published examples of transparent reporting. The STROBE checklist is best used in conjunction with this article (freely available on the Web sites of PLoS Medicine at http://www.plosmedicine.org/, Annals of Internal Medicine at http://www.annals.org/, and Epidemiology at http://www.epidem.com/). Information on the STROBE Initiative is available at www.strobe-statement.org.

***Supplementary File S2:*** Employee survey components

see the reference list in the manuscript for the here linked sources

| Variables / Scores | Category / Scale | Items | Source |
|---|---|---|---|
| Wahrnehmung von COVID-19<br>Disease perception | 2 questions on 7-point Likert scale | Das neuartige Coronavirus ist für mich …<br>nah … weit entfernt *<br>The novel coronavirus to me feels …<br>Close to me … far away from me<br>langsam ausbreitend … schnell ausbreitend<br>Spreading slowly … spreading fast | [25,48] |
| Affektives Risiko<br>Affective risk perception | 3 questions on 7-point Likert scale | etwas, woran ich dauernd denke … etwas, woran ich fast nie denke *<br>Something I think about all the time … Something I almost never think about<br>Angsteinflößend … nicht angsteinflößend *<br>Fear-inducing … not fear-inducing<br>Besorgnis erregend … nicht Besorgnis erregend *<br>Worrying … not worrying | [25,48] |
| Medien-Hype<br>Perceived adequacy of media coverage | 1 question on 7-point Likert scale | Medial aufgeblasen … medial zu wenig beachtet *<br>Media hyped … not media hyped | [25,48] |
| Hilflosigkeit<br>Perceived helplessness | 1 question on 7-point Likert scale | Etwas, bei dem ich mich hilflos fühle … etwas, gegen das ich aktiv etwas tun kann *<br>The SARS-CoV-2 is something …<br>… that makes me feel helpless … I am able to combat with my own action | [25,48] |
| Neuartigkeit<br>Perceived novelty | 1 question on 7-point Likert scale | Neu … alt *<br>New … old | [25,48] |
| Wissenschaftlicher Kenntnisstand<br>Scientific knowledge about COVID-19 | 1 question on 7-point Likert scale | Völlig unerforscht … sehr gut erforscht *<br>Completely unexplored … fully explored | [25,48] |
| Subjektive Anfälligkeit<br>Perceived personal susceptibility | 7-point Likert Scale | Als wie anfällig schätzen Sie sich für eine Infektion mit dem neuartigen Coronavirus ein?<br>How susceptible do you consider yourself to an infection with the novel coronavirus?<br>Überhaupt nicht anfällig … sehr anfällig<br>Not at all susceptible … very susceptible | [25,48] |
| Gefährlichkeit einer Infektion<br>Expected severity of infection with SARS-CoV-2 | 7-point Likert Scale | Wie schätzen Sie eine Infektion mit dem neuartigen Coronavirus für sich selbst ein?<br>How severe would contracting the novel coronavirus be for you?<br>Völlig harmlos ... extrem gefährlich<br>*Not severe … very severe* | [25,48] |
| Wahrscheinlichkeit einer Infektion im privaten Umfeld<br>Perceived probability of contracting COVID-19 in private surroundings | 7-point Likert Scale | Wie hoch schätzen Sie Ihre Wahrscheinlichkeit ein, dass Sie sich mit dem neuartigen Coronavirus *im Privatleben, also außerhalb der Arbeit,* infizieren?<br>What do you consider to be your own probability of getting infected with the novel coronavirus in your private surrounding?<br>Extrem unwahrscheinlich ... extrem wahrscheinlich<br>Extremely unlikely … extremely likely | [25,48] * |
| Zugehörigkeit zur Risikogruppe | Yes/no/not specified | Das Robert Koch-Institut nennt verschiedene Vorerkrankungen, die das Risiko eines schweren COVID-19-Krankheitsverlaufs (durch das Coronavirus ausgelöst) erhöhen. Zu diesen Erkrankungen zählen beispielsweise Herzkreislauferkrankungen, Diabetes, | [52] |

| | | | |
|---|---|---|---|
| Affiliation to risk group for developing severe COVID-19 | | Erkrankungen des Atmungssystems, der Leber und der Niere sowie Krebserkrankungen und Erkrankungen, die mit einer Immunschwäche einhergehen.<br>The Robert Koch Institute lists various pre-existing conditions or risk factors that were shown to be associated with an increased risk of developing severe COVID-19. An increased risk was assumed for individuals with coronary heart disease, diabetes mellitus, bronchial asthma or chronic bronchitis, liver disease, kidney problems, cancer, and diseases associated with immunodeficiency.<br><br>Zählen Sie aufgrund von Vorerkrankungen zur Risikogruppe für schwere COVID-19-Krankheitsverläufe?<br>Based on those criteria regarding pre-existing conditions, are you at increased risk of developing severe COVID-19? | |
| Umfeld mit Personen aus der Risikogruppe<br>Frequent contact with individuals from the risk group | Multiple choice; yes if at least one item=yes | Haben Sie regelmäßig nahen Kontakt (<1,50m) zu folgenden Personengruppen, die im Rahmen der COVID-19-Pandemie als Risikogruppen gelten?<br>Do you have regular close contact (<1.50m) with any individual at increased risk of developing severe COVID-19?<br>Personen, die aufgrund von Alter und/oder Vorerkrankungen zur Risikogruppe gehören.<br>Individuals belonging to the risk group due to age and/or pre-existing conditions<br>Personen, die Sie im Pflegeheim, Krankenhaus, oder in einer Reha-Einrichtung besuchen.<br>Individuals you are paying a visit in nursing homes, hospitals, or rehabilitation clinics.<br>Personen, die von einem ambulanten Pflegedienst betreut werden.<br>Individuals receiving outpatient care. | self-developed |
| Infizierte Personen im näheren Umfeld<br>Infected peers | Multiple choice | Bitte denken Sie nun an Ihr direktes Umfeld (privat und beruflich) im Zusammenhang mit dem neuartigen Coronavirus. Welche der Aussagen treffen auf Ihr persönliches Umfeld (privat und beruflich) zu?<br>Do you know people in your immediate social environment (private and at work) who are or have been infected with the novel coronavirus?<br>… Es gibt ungetestete Verdachtsfälle<br>… Yes, suspected but not yet confirmed by a test<br>… Es gibt bestätigte Fälle erkrankter Personen<br>… Yes, confirmed.<br>… Es gibt genesene Personen<br>… recovered from infection.<br>… Es gibt verstorbene Personen<br>… deceased due to infection.<br>… Es gibt keine Fälle<br>… No.<br>… Weiß nicht<br>… Don’t know. | [25,48] * |
| SARS-CoV-2 Schnelltest<br>SARS-CoV-2 Rapid antigen test | Yes/no/not specified | Haben Sie schon einmal einen Schnell-Test (Rachen-/Nasen-Abstrich) machen lassen, weil Sie Erkrankungssymptome (z.B. Fieber, Husten, Durchfall) oder Kontakt zu einer (möglicherweise) infizierten Person hatten oder in einem Risikogebiet waren?<br>Have you already performed a voluntary COVID-19 rapid test because of having been in close contact with a suspected or confirmed infected person, having been in a risk area, or showing cold symptoms (e.g., fever, cough)? | self-developed |
| Infektionsstatus<br>COVID-19 infection status | Single choice | Haben oder hatten Sie sich mit dem neuartigen Coronavirus infiziert?<br>Are you, or have you been, infected with the novel coronavirus?<br>… Ja, bestätigt und bereits überstanden<br>… yes, confirmed and recovered<br>… Ja, bestätigt und noch nicht überstanden<br>… yes, confirmed and not yet recovered. | [25,48] * |

| | | | |
|---|---|---|---|
| | | … Ja, aber noch nicht bestätigt<br>… yes, but not yet confirmed.<br>… Nein<br>… No.<br>… Weiß nicht<br>… Don’t know.<br>… Keine Angabe<br>… Not specified | |
| Antikörpertest<br>SARS-CoV-2-specific antibody test | Yes/no/not specified | Haben Sie bereits einen Test auf Antikörper gegen das neuartige Coronavirus machen lassen? (Blutuntersuchung)<br>Have you already performed a SARS-CoV-2-specific antibody test? | self-developed |
| Ergebnis des Antikörpertests<br>SARS-CoV-2-specific antibody test result | Single choice | Mein Testergebnis auf Antikörper war<br>Bei mehreren Tests bitte das aktuellste Testergebnis angeben.<br>The result of my latest antibody test was ...<br>… negativ (kein Hinweis auf eine durchgemachte Infektion)<br>… negative (no evidence of past infection)<br>… grenzwertig<br>… borderline<br>… positiv (möglicher Hinweis auf eine durchgemachte Infektion)<br>… positive (evidence of past infection)<br>… Keine Angabe<br>… not specified | self-developed |
| Durchführende Institution des Antikörpertests<br>Institute performing the SARS-CoV-2-specific antibody test | Multiple choice + free text | Wer hat den oder die Coronavirus-Antikörper-Test(s) (Blutuntersuchung) durchgeführt?<br>Who performed the antibody test (blood testing)?<br>… Betriebsarzt/ Betriebsärztin MED / occupational physician<br>… Hausarzt/Hausärztin / general practitioner<br>… Anderer Facharzt/Fachärztin / other medical specialist<br>… Eigentest (z.B. Apothekentest) / self test (e.g., pharmacy test)<br>… Sonstige: … / other, please specify … | self-developed |
| Corona-Warn-App des RKIs<br>RKI Corona-Warn-App | Yes/no/not specified | Nutzen Sie die neue Corona-Warn-App des Robert-Koch Instituts?<br>Do you use the Corona Warn App provided by the Robert Koch Institute? | self-developed |
| Nicht-Nutzung der Corona-Warn-App<br>Corona-Warn-App non-usage | Free text | Warum nutzen Sie die Corona-Warn-App nicht?<br>Why don’t you use the Corona Warning App? | self-developed |
| Corona-spezifische Resilienz<br>COVID-19-specific resilience | 4 questions on 7-point Likert Scale | Bitte beachten Sie die folgenden Fragen im Hinblick auf Ihre Erfahrungen in der Corona-Pandemie.<br>How do you feel during the COVID-19 pandemic?<br>Bitte geben Sie an, inwiefern Sie den einzelnen Aussagen zustimmen.<br>Please indicate to what extent you agree with each statement listed below.<br>Stimme überhaupt nicht zu … Stimme voll und ganz zu<br>… strongly disagree … strongly agree<br>…finde ich die für mich notwendigen Wege, um weiterzumachen.<br>… I find ways to keep going. | [25,48] |

| | | | |
|---|---|---|---|
| | | … weiß ich, dass ich mich nicht unterkriegen lasse.<br>… I know that I can get through hard times. | |
| | | …lerne ich wichtige und nützliche Lektionen für mein Leben.<br>… I learn important lessons for life. | |
| | | …erlerne ich Möglichkeiten, beim nächsten Mal besser damit umzugehen.<br>… I am learning ways to cope better next time | |
| Arbeitssituation vor der Coronavirus-Pandemie<br>Workplace location before the COVID-19 pandemic | Percentage rating + free text | Im Folgenden möchten wir Sie bitten, Ihre Arbeitssituation zu zwei Zeitpunkten einzuschätzen: wie war es VOR der Coronavirus-Pandemie und wie erleben Sie Ihre Arbeitssituation AKTUELL?<br>In the following, we ask you to assess your work situation at two time points: How did you experience your work situation BEFORE the COVID-19 pandemic, and how do you feel CURRENTLY?<br>Der Zeitraum „vor-Corona“ meint Ihre normale Arbeitssituation, so wie sie vor der Coronavirus-Pandemie war.<br>Der Zeitraum „aktuell“ meint ihre jetzige Arbeitssituation, so wie Sie sie derzeit erleben.<br>Bitte bewerten Sie die gleichen Aussagen jeweils aus diesen beiden Perspektiven.<br>The time "BEFORE the COVID-19 pandemic" describes your usual work situation before the COVID-19 pandemic, "CURRENTLY" describes your current work situation.<br>Please rate each statement from these two perspectives.<br>Mit welchen Anteilen (%) haben Sie vor der Corona-Pandemie an folgenden Orten gearbeitet?<br>Hierbei sind maximal 100 % möglich: z.B. 50 % im Betrieb und 50 % im Außendienst.<br>Before the COVID-19 pandemic, what percent of your working time did you spend working from the following locations?<br>100 % is the maximum possible: e.g., 50 % on-site, 50 % in external sales.<br>… vor Ort im Betrieb<br>… on-site<br>… im Außendienst (z.B. Einkauf, Verkauf, vor Ort beim Kunden)<br>… in external sales (e.g., purchasing, sales, on-site at the customer)<br>… in Telearbeit (Remote-Work) von Zuhause<br>.. remote<br>… Sonstige: … / other, please specify … | [45] |
| Arbeitssituation während der Coronavirus-Pandemie<br>Workplace location during COVID-19 pandemic | Percentage rating + free text | Mit welchen Anteilen (%) arbeiten Sie aktuell (in der letzten Woche) an folgenden Orten?<br>How many parts of your working time do you currently (last week) spend …<br>100 % is the maximum possible: e.g., 50 % on-site, 50 % in external sales.<br>… vor Ort im Betrieb<br>… on-site<br>… im Außendienst (z.B. Einkauf, Verkauf, vor Ort beim Kunden)<br>… in external sales (e.g., purchasing, sales, on-site at the customer)<br>… in Telearbeit (Remote-Work) von Zuhause<br>.. remote<br>… Sonstige: … / other, please specify … | self-developed |
| Arbeitsinhalte<br>Work content | Before and during pandemic: 3 questions on 5-point Likert Scale | Weiter geht es nun mit verschiedenen Aspekten am Arbeitsplatz, die sowohl Belastungen als auch Unterstützungsmöglichkeiten in den Blick nehmen.<br>Please score the following stress and strain factors and resources compared to before and during the COVID-19 pandemic.<br>Wie viel Einfluss haben Sie darauf, welche Arbeit Ihnen zugeteilt wird?<br>How much can you influence what work is assigned to you?<br>Gar keinen Einfluss … sehr hohen Einfluss | [43,44,45] |

| | | | |
|---|---|---|---|
| | | … Vor der Pandemie<br>… Aktuell<br>Before the COVID-19 pandemic: Not at all … very much …<br>During the COVID-19 pandemic: Not at all … very much … | |
| | | Wenn Sie Ihre Tätigkeit insgesamt betrachten, inwieweit können Sie die Reihenfolge der Arbeitsschritte selbst bestimmen?<br>To what extent can you determine the order of the work steps yourself?<br>Gar keinen Einfluss … sehr hohen Einfluss<br>… Vor der Pandemie<br>… Aktuell<br>Before the COVID-19 pandemic: Not at all … very much …<br>During the COVID-19 pandemic: Not at all … very much … | |
| | | Oft fehlen mir die benötigten Informationen, Materialien und Arbeitsmittel. *<br>I often lack the information, materials and tools I need. *<br>Trifft gar nicht zu … Trifft völlig zu<br>… Vor der Pandemie<br>… Aktuell<br>Before the COVID-19 pandemic: Strongly disagree … fully agree …<br>During the COVID-19 pandemic: Strongly disagree … fully agree … | |
| Arbeitsorganisation<br>Organisation of work | Before and during a pandemic: 3 questions on a 5-point Likert Scale | Ich habe zu viel Arbeit. *<br>I have too much work to do. *<br>Trifft gar nicht zu … Trifft völlig zu<br>… Vor der Pandemie<br>… Aktuell<br>Before the COVID-19 pandemic: Strongly disagree … fully agree …<br>During the COVID-19 pandemic: Strongly disagree … fully agree … | [43,44,45] |
| | | Ich stehe häufig unter Zeitdruck. *<br>I often feel like I am under time pressure. *<br>Trifft gar nicht zu … Trifft völlig zu<br>… Vor der Pandemie<br>… Aktuell<br>Before the SARS-CoV-2 pandemic: Strongly disagree … fully agree …<br>During the SARS-CoV-2 pandemic: Strongly disagree … fully agree … | |
| | | Ich werde bei meiner Arbeit immer wieder unterbrochen (beispielsweise durch andere Personen, das Telefon, etc.). *<br>I am interrupted in my work constantly (e.g. by other people, the telephone, etc.). *<br>Trifft gar nicht zu … Trifft völlig zu<br>… Vor der Pandemie<br>… Aktuell<br>Before the COVID-19 pandemic: Strongly disagree … fully agree …<br>During the COVID-19 pandemic: Strongly disagree … fully agree … | |
| Arbeitsumgebung<br>Working environment | Before and during pandemic: 3 questions on 5-point Likert Scale | An meinem Arbeitsplatz gibt es ungünstige Umgebungsbedingungen, wie Lärm, Klima, Staub. *<br>There are unfavorable environmental conditions at my workplace, e.g., noise, climate, and dust. *<br>Trifft gar nicht zu … Trifft völlig zu<br>… Vor der Pandemie | [43,44,45] |

| | | | |
|---|---|---|---|
| | | … Aktuell<br>Before the COVID-19 pandemic: Strongly disagree … fully agree …<br>During the COVID-19 pandemic: Strongly disagree … fully agree … | |
| | | An meinem Arbeitsplatz sind Räume und Raumausstattung ungenügend. *<br>The rooms and room setup are inadequate at my workplace. *<br>Trifft gar nicht zu … Trifft völlig zu<br>… Vor der Pandemie<br>… Aktuell<br>Before the COVID-19 pandemic: Strongly disagree … fully agree …<br>During the COVID-19 pandemic: Strongly disagree … fully agree … | |
| | | An meinem Arbeitsplatz ist die Ausstattung mit Arbeitsmitteln unzureichend (z.B. fehlendes/ungeeignetes Werkzeug, ungünstige Bedienung oder Einrichtung von Maschinen, unzureichende Softwaregestaltung). *<br>The equipment at my workplace is inadequate (e.g. missing/unsuitable tools, unfavorable operation or setup of machines, inadequate software design). *<br>Trifft gar nicht zu … Trifft völlig zu<br>… Vor der Pandemie<br>… Aktuell<br>Before the COVID-19 pandemic: Strongly disagree … fully agree …<br>During the COVID-19 pandemic: Strongly disagree … fully agree … | |
| | | Ich kann mich auf meine Kollegen/Kolleginnen verlassen, wenn es bei der Arbeit schwierig wird.<br>I can rely on my colleagues when dealing with difficulties at work.<br>Trifft gar nicht zu … Trifft völlig zu<br>… Vor der Pandemie<br>… Aktuell<br>Before the COVID-19 pandemic: Strongly disagree … fully agree …<br>During the COVID-19 pandemic: Strongly disagree … fully agree … | |
| Soziale Beziehungen auf der Arbeit<br>Social relations at work | Before and during pandemic: 3 questions on 5-point Likert Scale | Ich kann mich auf meine/n direkte/n Vorgesetzte/n verlassen, wenn es bei der Arbeit schwierig wird.<br>I can relate on my leader, when dealing with difficulties at work.<br>Trifft gar nicht zu … Trifft völlig zu<br>… Vor der Pandemie<br>… Aktuell<br>Before the COVID-19 pandemic: Strongly disagree … fully agree …<br>During the COVID-19 pandemic: Strongly disagree … fully agree … | [43,44,45] |
| | | Ich bekomme von Vorgesetzten und Kollegen/Kolleginnen ausreichend Rückmeldung über die Qualität meiner Arbeit.<br>I regularly receive feedback about the quality of my work.<br>Trifft gar nicht zu … Trifft völlig zu<br>… Vor der Pandemie<br>… Aktuell<br>Before the COVID-19 pandemic: Strongly disagree … fully agree …<br>During the COVID-19 pandemic: Strongly disagree … fully agree … | |
| Haltung zu empfohlenen technischen Schutz- und | 10 questions on 5-point Likert Scale | Für wie geeignet halten Sie die folgenden empfohlenen Schutz- und Hygienemaßnahmen am Arbeitsplatz, um einer Infektion mit dem neuartigen Coronavirus vorzubeugen? | self-developed based on [12] |

| | | | |
|---|---|---|---|
| Hygienemaßnahmen am Arbeitsplatz<br>Attitude toward recommended technical safety and health measures for infection control | | How appropriate do you consider the following recommended measures to prevent the spread and infection of the novel coronavirus in the workplace? Please evaluate all preventive measures on a technical level listed below.<br>Not appropriate at all ... very appropriate<br>Gar nicht geeignet … sehr gut geeignet<br>i. Sicherheitsabstand am Arbeitsplatz einhalten (mind. 1,5m)<br>Keep distance from other people / Maintain a distance of at least 1.5 m at the workplace<br>ii. Regelmäßiges Lüften von Arbeitsräumen<br>Ventilate rooms regularly<br>iii. Aufstellen von Kontakt-/Spuckschutz am Arbeitsplatz<br>Protection of employees with, for example, Plexiglas planes<br>iv. Räumliche Gestaltung der Arbeitsplätze, um ausreichenden Abstand zu gewährleisten<br>A distance of at least 1.5m between the individual workstations must be maintained, and workplaces should be arranged accordingly.<br>v. Bildung von festen, nicht wechselnden Teams am Arbeitsplatz<br>Form fixed teams in order to reduce mixing<br>vi. Wenn möglich, Nutzung von Home-Office-Regelungen<br>Home office, if possible<br>vii. Wenn möglich, Durchführung von Online-Besprechungen, z.B. über Skype<br>Meetings are preferably held online/ via Skype<br>viii. Besprechungen nur in Räumen durchführen, wenn Abstandsregel (1,5m) eingehalten werden kann<br>Use large rooms to ensure a distance of 1.5m<br>ix. Nutzung der Kantine reduzieren<br>Reduce spent time at the canteen or on-site stores, especially during peak hours<br>x. Regelmäßige Reinigung (z.B. Büroräume, Teeküche, Produktionsumfeld, Werkstätten, Verkehrswege etc.)<br>Perform frequent cleaning of offices, meeting rooms, kitchenettes, traffic routes, sanitary facilities, etc. | |
| Haltung zu empfohlenen organisatorischen Schutz- und Hygienemaßnahmen am Arbeitsplatz<br>Attitude toward recommended organisational safety and health measures for infection control | 6 questions on 5-point Likert Scale | Für wie geeignet halten Sie die folgenden empfohlenen Schutz- und Hygienemaßnahmen am Arbeitsplatz, um einer Infektion mit dem neuartigen Coronavirus vorzubeugen?<br>How appropriate do you consider the following recommended measures to prevent the spread and infection of the novel coronavirus in the workplace? Please evaluate all preventive measures on an organisational level listed below.<br>Not appropriate at all ... very appropriate<br>Gar nicht geeignet … sehr gut geeignet<br>i. Feste Zuordnung der Mitarbeitenden zu einem Arbeitsplatz<br>Define personally assigned workstations, if possible<br>ii. Zuordnung von Arbeitsmitteln für bestimmte Personen (z.B. Maus/Tastatur/Werkzeug)<br>Define personally assigned work equipment (e.g., mouse/ keyboard)<br>iii. Zusammentreffen (Gruppenbildung) von mehreren Beschäftigten am Arbeitsplatz vermeiden (z.B. falls möglich: telefonischer Schichtwechsel)<br>Avoid group gatherings (e.g., shift information transfer via electronic media, where possible)<br>iv. Unnötigen Durchgangsverkehr in stark frequentierten Bereichen vermeiden (z.B. in Büros, Hallen, Treppenhaus)<br>Avoid unnecessary through traffic in highly frequented areas (e.g., offices, halls) | self-developed based on [12] |

| | | | |
|---|---|---|---|
| | | v. Pausen zeitlich versetzt abhalten<br>Break times are decoupled<br>vi. Überwachung der Einhaltung der Hygieneregeln durch den/die Vorgesetzte/n<br>Supervision of compliance with the hygiene rules by supervisor | |
| Haltung zu empfohlenen persönlichen Schutz- und Hygienemaßnahmen am Arbeitsplatz<br>Attitude toward recommended personal safety and health measures for infection control | 10 questions on 5-point Likert Scale | Für wie geeignet halten Sie die folgenden empfohlenen Schutz- und Hygienemaßnahmen am Arbeitsplatz, um einer Infektion mit dem neuartigen Coronavirus vorzubeugen?<br>Gar nicht geeignet … sehr gut geeignet<br>How appropriate do you consider the following recommended measures to prevent the spread and infection of the novel coronavirus in the workplace? Please evaluate all preventive measures on an organisational level listed below.<br>Not appropriate at all ... very appropriate<br>i. Regelmäßig Hände waschen oder desinfizieren<br>Wash hands thoroughly with soap and water regularly<br>ii. Vermeiden von Händeschütteln<br>Avoid personal greetings by shaking hands / avoid shaking hands<br>iii. Beachten von richtiger Hust- und Niesetikette<br>Cough into your sleeve and turn away from others when coughing<br>iv. Tragen einer Mund-Nasen-Bedeckung<br>Mouth-nose-covers (MNC) must be worn<br>v. Mit Krankheitssymptomen zu Hause bleiben<br>Employees with cold symptoms and/ or fever are to stay at home<br>vi. Auf dem Weg zur Arbeit bevorzugt Individualtransport nutzen<br>Preferably use your personal vehicle or other personal form of transportation<br>vii. Beschäftigte, die zu Risikogruppen für schwere Verläufe gehören, besonders schützen<br>Special measures to protect employees at high risk are introduced<br>viii. Beratung durch Betriebsarzt/Betriebsärztin ermöglichen<br>Consulting by the on-site doctor or MED<br>ix. Aufstellen von Hinweisschildern mit Informationen zu Hygiene- und Verhaltensregeln am Arbeitsplatz<br>Install informational signs covering hygiene and rules of conduct at the workplace<br>x. Sicherheitsunterweisungen zu Hygiene- und Verhaltensregeln am Arbeitsplatz<br>A communication guide for employees has been prepared (hygiene and conduct rules) and posted in highly frequented areas | self-developed based on [12] |
| Reaktanz<br>COVID-19 specific reactance | 4 questions on 7-point Likert Scale | Denken Sie nun an die aktuell in Ihrem Unternehmen ergriffenen Maßnahmen, die verhindern sollen, dass sich das neuartige Coronavirus weiter ausbreitet.<br>Überhaupt nicht … sehr<br>Imagine the current situation of introduced SARS-Cov-2-infection control measures in the workplace.<br>Not at all ... very much<br>i. Wie sehr empfinden Sie die Maßnahmen als Freiheitseinschränkung?<br>Do you perceive the measures as restricting your freedom?<br>ii. Wie sehr stören Sie die Maßnahmen?<br>How disturbed do you feel by the measures? | [25,48] * |

| | | | |
|---|---|---|---|
| | | iii. Wie sehr frustriert sind Sie über die Maßnahmen?<br>How frustrated do you feel about the measures? | |
| | | iv. Wie sehr ärgern Sie die Maßnahmen?<br>How annoyed do you feel about the measures? | |
| Einhaltung von Abstandsregeln durch Kollegen<br>Colleagues adherence to distance rule | 7-point Likert Scale | Ich traue meinen Kollegen/Kolleginnen zu, sich am Arbeitsplatz an die Abstandsregeln zu halten.<br>Gar nicht … immer<br>I trust my colleagues to adhere to the distance rules in the workplace.<br>Not at all … always | [25,48] * |
| Einhaltung von Hygieneregeln durch Kollegen<br>Colleagues' adherence to hygiene rules | 7-point Likert Scale | Ich traue meinen Kollegen/Kolleginnen zu, sich am Arbeitsplatz an die Hygieneregeln zu halten.<br>Gar nicht … immer<br>I trust my colleagues to adhere to the hygiene rules in the workplace.<br>Not at all … always | [25,48] * |
| Einhaltung von Verhaltensregeln durch Kollegen<br>Colleagues' adherence to norm use | Yes/no/have not been in that situation | Haben Sie in der letzten Woche auf andere Kollegen/Kolleginnen eingewirkt, sich an die empfohlenen Verhaltensregeln zu halten, wenn diese nicht eingehalten wurden (z.B. auf erforderliche Abstände hingewiesen)?<br>Have you encouraged other colleagues to adhere to the recommended rules of conduct if they were not followed within the last week (e.g. pointed out necessary distances)? | [25,48] * |
| Krankenstand<br>Sick leave | Yes/no/not specified | Haben Sie den Eindruck, dass sich der Krankenstand in Ihrem Betrieb während der Corona-Pandemie erhöht hat?<br>Do you have the impression that sick leave in your company has increased during the COVID-19 pandemic? | self-developed |
| Wahrscheinlichkeit einer Infektion am Arbeitsplatz<br>Perceived probability of contracting COVID-19 in the workplace | 7-point Likert Scale | Wie hoch schätzen Sie die Wahrscheinlichkeit ein, dass Sie sich mit dem neuartigen Coronavirus am Arbeitsplatz infizieren?<br>What do you consider to be your own probability of getting infected with the novel coronavirus in your workplace?<br>Extrem unwahrscheinlich .. extrem wahrscheinlich<br>Extremely unlikely … extremely likely | [25,48] * |
| Möglichkeiten der Vermeidung einer Infektion am Arbeitsplatz<br>Perceived self-efficacy | 7-point Likert Scale | In der jetzigen Situation eine Infektion mit dem neuartigen Coronavirus am Arbeitsplatz zu vermeiden, ist für mich...<br>For me, avoiding an infection with the novel coronavirus in the current situation in the workplace is …<br>… extrem schwierig … extrem einfach<br>… extremely difficult … extremely easy | [25,48] * |
| Informiertsein<br>Being informed about potential infection risks in the workplace | 5-point Likert Scale and not specified | Wie gut fühlen Sie sich insgesamt über Gefährdungen und Gesundheitsrisiken informiert, die im Zusammenhang mit dem neuartigen Coronavirus durch Ihre Arbeit bzw. an Ihrem Arbeitsplatz entstehen können?<br>How well informed do you feel about any dangers and health risks that may arise concerning the novel coronavirus through your work or in your workplace?<br>Mangelhaft … sehr gut<br>Inadequately … very good | [42] * |
| Bewertungs des Arbeitsschutzengagements des Arbeitsgebers<br>Employees' rating of the employer's commitment to occupational safety and health | 4-point Likert Scale | Wie würden Sie insgesamt betrachtet das Arbeitsschutzengagement in Bezug auf das neuartige Coronavirus in Ihrem Unternehmen bewerten? Sehr gering … sehr hoch<br>How do you rate the commitment of your employer to occupational safety and health strategy to prevent the transmission of SARS-Cov-2?<br>Very low … very high | [42] * |
| Bundesland der Arbeit<br>Federal state of work | Single choice | In welchem Bundesland arbeiten Sie?<br>In which German federal state do you work?<br>List of all 16 German states | [25,48] * |

| | | | |
|---|---|---|---|
| Standort<br>Company site of work | Single choice + free text | An welchem Standort arbeiten Sie? List of 6 items + other<br>At which company site do you work? | self-developed |
| Tätigkeitsbereich<br>Professional activity | Single choice + free text | In welchem Bereich sind Sie vor allem tätig?<br>In which area are you (primarily) employed?<br>… Großraumbüro open-plan office<br>… Enge Montagelinie assembly line with confined spaces<br>… Werkschutz factory security service<br>… Werkärztlicher Bereich company medical service<br>… Sonstige: … other: … | [38] |
| Berufliche Ausbildung<br>Professional education | Single choice | Welche Ausbildung wird üblicherweise für Ihre jetzige Tätigkeit benötigt?<br>Which professional education is typically required for your current job?<br>… Keine Berufsausbildung None<br>… Abgeschlossene Berufsausbildung ohne Zusatzqualifikation Completed professional education without additional qualifications<br>… Abgeschlossene Berufsausbildung mit Zusatzqualifikation Completed professional education with additional qualifications<br>… Hochschul-/Fachhochschulstudium: Studium mit weniger als 4 Jahren Regelstudienzeit (Bachelor, Diplom an einer Verwaltungsfachhochschule) University/technical college studies: less than 4 years of study (bachelor, diploma at a business school)<br>… Studium mit mindestens 4 Jahren Regelstudienzeit (Diplom, Master, Magister, Staatsprüfung, Lehramtsprüfung) at least 4 years of study (diploma, Master, Magister, exam, teacher’s examination)<br>… Trifft nicht zu, da Auszubildende/-r<br>… not applicable, as I am a trainee | [40] |
| Berufserfahrung<br>Work experience in years | Number | Seit wie vielen Jahren arbeiten Sie in Ihrem Beruf?<br>How many years have you worked within your profession?<br>… Seit weniger als einem Jahr For less than one year<br>… Seit Anzahl Jahren: … For … years | [47] * |
| Unternehmens-zugehörigkeit<br>Employment at company in years | Number | Seit wie vielen Jahren arbeiten Sie bei Ihrem jetzigen Arbeitgeber?<br>How many years have you worked at the current company?<br>… Seit weniger als einem Jahr For less than one year<br>… Seit Anzahl Jahren: … For … years | [47] * |
| Führungsposition<br>Leadership position | Yes/no | Sind Sie für andere Beschäftigte der/die Vorgesetzte?<br>Are you the supervisor for other employees? | [38] |
| Befristeter Arbeitsvertrag<br>Fixed-term contract | Yes/no | Haben Sie einen befristeten Arbeitsvertrag?<br>Do you have a fixed-term contract? | [38] |
| Vollzeit Tätigkeit<br>Full-time job | Yes/no | Arbeiten Sie in Vollzeit?<br>Do you work full time? | [38] |
| Kurzarbeit<br>Short-time work | Yes/no | Sind Sie derzeit in Kurzarbeit?<br>Do you currently work short-time? | self-developed |
| Schichtarbeit<br>Shift work | Yes/no | Arbeiten Sie im Schichtdienst?<br>Do you work in shifts? | [38] |
| Tätigkeit in wechselnden Teams<br>Work in changing teams | Yes/no | Arbeiten Sie in immer wieder wechselnden Teams?<br>Do you work in constantly changing teams? | self-developed |
| Zeitarbeit<br>Temporary work | Yes/no | Arbeiten Sie in Zeitarbeit?<br>Do you work in temporary work? | self-developed |

| | | | |
|---|---|---|---|
| Alter<br>Age | Number | Wann sind Sie geboren?<br>In what year were you born? | [40] |
| Geschlecht<br>Gender | Male/female/diverse | Welches Geschlecht haben Sie?<br>What is your gender? | [38] |
| Staatsangehörigkeit<br>Nationality | 2 questions<br>Yes/no/not specified | Haben Sie die deutsche Staatsangehörigkeit?<br>Are you German citizen? | [41] * |
| | | Haben Sie zusätzlich eine andere Staatsangehörigkeit?<br>Do you also have another citizenship? | |
| Höchste Schulbildung<br>School-leaving certificate | Single choice | Welchen höchsten Schulabschluss haben Sie? Ordnen Sie bitte im Ausland erworbene Abschlüsse einem gleichwertigen deutschen Abschluss zu.<br>What is your highest school degree? Please match qualifications obtained abroad with the German equivalent.<br>… Abschluss nach höchstens 7 Jahren Schulbesuch<br>… up to 7 years of school education<br>… Haupt-/Volksschulabschluss<br>… up to years of school education<br>… Polytechnische Oberschule der DDR mit Abschluss der 8. oder 9. Klasse<br>… Polytechnic high school of the GDR with completion of the 8th or 9th grade<br>… Polytechnische Oberschule der DDR mit mit Abschluss der 10. Klasse<br>… Polytechnic high school of the GDR with completion of the 10th grade<br>… Realschulabschluss, Mittlere Reife oder gleichwertiger Abschluss<br>… 10 years and more without general qualification for university entrance<br>… Fachhochschulreife<br>… advanced technical college certificate<br>… Abitur (Allgemeine oder fachgebundene Hochschulreife)<br>… General university entrance qualification, international baccalaureate<br>… Sonstiger Schulabschluss: … Other … | [40] |
| Bundesland Wohnen<br>Federal state of residence | Single choice | In welchem Bundesland wohnen Sie? List of all 16 German states<br>In which German state do you live? | [25,48] |
| Größe des Haushalts<br>Number of household members | Single choice | Wie viele Personen leben ständig in Ihrem Haushalt?<br>How many people live in your household?<br>… Nur ich<br>… I live alone<br>... 2 Personen<br>… Two individuals in the same household<br>… 3 bis 4 Personen<br>… Three to four individuals in the same household<br>… Mehr als 4 Personen<br>… More than four individuals in the same household<br>… Keine Angabe | [40] * |
| Gesundheitsfachkraft im Haushalt | Yes/no/not specified | Übt eine Person in Ihrem Haushalt einen Beruf im Gesundheitssektor aus?<br>Does a person living in your household work as a health professional? | [25,48] * |

| | | | |
|---|---|---|---|
| Presence of health professional within household | | | |
| Beziehungsstatus<br>Living in a committed relationship | Yes/no/not specified | Leben Sie in fester Partnerschaft?<br>Do you live in a committed relationship or marriage? | [25,48] * |
| Kinder unter 18 J<br>Children younger than 18 y | Yes/no/not specified | Haben Sie ein oder mehrere Kinder unter 18 Jahren, die in Ihrem Haushalt leben?<br>Do you have children under 18 years living at home with you? | self-developed |
| Betreuungssituation der Kinder<br>Child care | 2 questions on 7-point Likert Scale | Wie sehr stimmen Sie den folgenden Aussagen in der aktuellen Situation zu?<br>Do you agree with the following statements in the current situation?<br>Überhaupt nicht … sehr<br>Not at all … very<br>… Der Unterricht und/oder die Betreuung meiner Kinder werden in einem guten Maße weiter umgesetzt.<br>… Teaching and/or care of my children continues to be implemented appropriately. | [25,48] |
| | | ... Die Betreuung meiner Kinder belastet mich zusätzlich.<br>… The care of my children is an additional burden for me. | |
| Freizeit<br>Leisure time | Multiple choice + free text | Sind Sie in der letzten Woche folgenden Freizeitaktivitäten nachgegangen?<br>Have you performed any of the following leisure activities within the past week?<br>Reisen in andere Bundesländer<br>Travelled to other German states | self-developed |
| | | Reisen in andere Länder<br>Travelled to other countries | |
| | | Gruppensportarten (z.B. Fußball, Volleyball)<br>Performed sports activities (e.g., soccer, volleyball) | |
| | | Sonstiges: …<br>other, please specify: … | |
| Big-Five Traits:<br>Persönlichkeitstypen<br>Extraversion<br>Neurozitismus<br>Offenheit<br>Verträglichkeit<br>Gewissenhaftigkeit<br>Neurocitism<br>Extraversion<br>Openness<br>Agreeableness<br>Conscientiousness | 10 questions on 5-point Likert Scale | Bitte geben Sie zu jeder Aussage Ihre spontane Einschätzung ab. Inwieweit treffen folgende Aussagen auf Sie zu?<br>How well do the following statements describe your personality? I see myself as someone who…<br>Trifft überhaupt nicht zu … Trifft voll und ganz zu<br>Disagree strongly … agree strongly<br>Ich bin eher zurückhaltend, reserviert. *<br>…is reserved * | [46] |
| | | Ich schenke anderen leicht Vertrauen, glaube an das Gute im Menschen.<br>…is generally trusting | |
| | | Ich bin bequem, neige zu Faulheit. *<br>…tends to be lazy * | |
| | | Ich bin entspannt, lasse mich durch Stress nicht aus der Ruhe bringen. *<br>…is relaxed, handles stress well * | |
| | | Ich habe nur wenig künstlerisches Interesse. *<br>…has few artistic interests * | |
| | | Ich gehe aus mir heraus, bin gesellig.<br>…is outgoing, sociable | |
| | | Ich neige dazu, andere zu kritisieren. *<br>…tends to find fault with others * | |

| | | | |
|---|---|---|---|
| | | Ich erledige Aufgaben gründlich.<br>…does a thorough job | |
| | | Ich werde leicht nervös und unsicher.<br>…gets nervous easily | |
| | | Ich habe eine aktive Vorstellungskraft, bin fantasievoll.<br>…has an active imagination | |
| Soziale Erwünschtheit<br>Social desirability | 6 questions on 5-point Likert Scale | Die folgenden Aussagen können auf Sie selbst mehr oder weniger zutreffen. Bitte geben Sie bei jeder Aussage an, wie sehr die Aussage auf Sie zutrifft.<br>The following statements may apply more or less to you personally. Please indicate to what extent they apply to you.<br>Trifft gar nicht zu … Trifft voll und ganz zu<br>Doesn't apply at all … applies completely<br>Im Streit bleibe ich stets sachlich und objektiv.<br>In an argument, I always remain objective and stick to the facts. | [39] |
| | | Auch wenn ich selbst gestresst bin, behandle ich andere immer freundlich und zuvorkommend.<br>Even if I am feeling stressed, I am always friendly and polite to others. | |
| | | Wenn ich mich mit jemandem unterhalte, höre ich ihm immer aufmerksam zu.<br>When talking to someone I always listen carefully to what the other person says. | |
| | | Es ist schon mal vorgekommen, dass ich jemanden ausgenutzt habe.<br>It has happened that I have taken advantage of someone in the past. | |
| | | Ich habe schon mal Müll einfach in die Landschaft oder auf die Straße geworfen.<br>I have occasionally thrown litter away in the countryside or on to the road. | |
| | | Manchmal helfe ich jemandem nur, wenn ich eine Gegenleistung erwarten kann.<br>Sometimes I only help people if I expect to get something in return. | |
| Anonymisierter Code<br>Anonymized code | Numbers / letters | Die beiden letzten Buchstaben des Nachnamens Ihrer Mutter: …<br>Last two letters of your mother's last name | |
| | | Die Anzahl der Buchstaben des (ersten) Vornamens Ihrer Mutter: …<br>Number of letters of your mother's first name | |
| | | Die beiden letzten Buchstaben des (ersten) Vornamens Ihres Vaters: …<br>Last two letters of your father's first name | |
| | | Ihr eigener Geburtstag (nur der Tag): …<br>The date (day only) of your own birthday in numbers | |
| *Note: Translated to English by the authors for the purpose of publication. The original version was in German only.* | | **recoded* | **adapted to the target group* |

***Supplementary File 3:*** Sensitivity analysis: results excluding observations with missing values

**Attitude toward technical occupational infection control measures**

| **Professional activity** | | **Office remote (n=332)** | | | **Office on-site (n=1299)** | | | **Assembly line and manufacturing (n=290)** | | |
|---|---|---|---|---|---|---|---|---|---|---|
| Block | Variables | Coef. | SE | p-value | Coef. | SE | p-value | Coef. | SE | p-value |
| Socio-demographics (I) | Gender (vs. male) | | | | | | | | | |
| | female | 0.15 | 0.06 | 0.007 ** | 0.08 | 0.03 | 0.015 * | - 0.28 | 0.11 | <0.010 ** |
| | Age (vs. 18-29 years) | | | | | | | | | |
| | 30-59 years | 0.09 | 0.09 | 0.290 | 0.04 | 0.04 | 0.337 | - 0.05 | 0.14 | 0.719 |
| | 60-67 years | 0.05 | 0.12 | 0.679 | 0.17 | 0.08 | 0.025 * | 0.25 | 0.27 | 0.343 |
| General workplace characteristics (II) | Shift work (vs. no) | | | | | | | | | |
| | Yes | - | - | - | - | - | - | - 0.14 | 0.10 | 0.151 |
| Perception regarding the pandemic-related impact in the workplace (III) | Reactance | - 0.08 | 0.02 | <0.001 *** | - 0.09 | 0.01 | <0.001 *** | - 0.08 | 0.03 | 0.022 * |
| | Information provided by employer | 0.07 | 0.03 | 0.021 * | 0.09 | 0.02 | <0.001 *** | 0.15 | 0.05 | 0.006 ** |
| | Trust in colleagues to adhere to distance rules | 0.03 | 0.02 | 0.117 | 0.07 | 0.01 | <0.001 *** | 0.12 | 0.03 | <0.001 *** |
| Employee's attitude toward COVID-19 in general (IV) | COVID-19-specific resilience | 0.09 | 0.03 | 0.002 ** | 0.10 | 0.02 | <0.001 *** | 0.15 | 0.04 | <0.001 *** |
| | Affective risk perception | 0.05 | 0.02 | 0.038 * | 0.11 | 0.01 | <0.001 *** | 0.09 | 0.04 | 0.008 ** |
| Log-like and DF | | - 203.9744 on 644 degrees | | | - 974.7564 on 2578 degrees | | | - 337.9526 on 560 degrees | | |

*p <0.05, **p <0.01, ***p <0.001

All models were controlled for social desirability, 7-day-incidence, company site, and educational background.
No Hauck-Donner effect was found.

**Attitude toward organisational occupational infection control measures**

| **Professional activity** | | **Office remote (n=332)** | | | **Office on-site (n=1299)** | | | **Assembly line and manufacturing (n=290)** | | |
|---|---|---|---|---|---|---|---|---|---|---|
| Block | Variables | Coef. | SE | p-value | Coef. | SE | p-value | Coef. | SE | p-value |
| Socio-demographics (I) | Gender (vs. male) | | | | | | | | | |
| | female | 0.29 | 0.08 | <0.001 *** | 0.12 | 0.05 | 0.008 ** | - 0.14 | 0.12 | 0.238 |
| | Age (vs. 18-29 years) | | | | | | | | | |
| | 30-59 years | 0.14 | 0.12 | 0.239 | 0.06 | 0.06 | 0.279 | 0.02 | 0.15 | 0.904 |
| | 60-67 years | 0.17 | 0.17 | 0.321 | 0.21 | 0.10 | 0.042 * | 0.62 | 0.30 | 0.039 * |
| General workplace characteristics (II) | Shift work (vs. no) | | | | | | | | | |
| | Yes | - | - | - | - | - | - | - 0.15 | 0.11 | 0.166 |
| Perception regarding the pandemic-related impact in the workplace (III) | Reactance | - 0.11 | 0.02 | <0.001 *** | - 0.10 | 0.01 | <0.001 *** | - 0.07 | 0.03 | 0.023 * |
| | Information provided by employer | 0.02 | 0.04 | 0.546 | 0.05 | 0.02 | 0.026 * | 0.03 | 0.06 | 0.592 |
| | Trust in colleagues to adhere to distance rules | 0.03 | 0.03 | 0.351 | 0.07 | 0.01 | <0.001 *** | 0.12 | 0.03 | <0.001 *** |
| Employee's attitude toward COVID-19 in general (IV) | COVID-19-specific resilience | 0.09 | 0.04 | 30.020 * | 0.09 | 0.02 | <0.001 *** | 0.15 | 0.05 | 0.002 ** |
| | Affective risk perception | 0.08 | 0.03 | 0.013 * | 0.16 | 0.02 | <0.001 *** | 0.10 | 0.04 | 0.007 ** |
| Log-like and DF | | - 303.0615 on 644 degrees | | | - 1324.772 on 2578 degrees | | | - 364.924 on 560 degrees | | |

*p <0.05, **p <0.01, ***p <0.001

All models were controlled for social desirability, 7-day-incidence, company site, and educational background.
No Hauck-Donner effect was found.

**Attitude toward personal occupational infection control measures**

| **Professional activity** | | **Office remote (n=332)** | | | **Office on-site (n=1299)** | | | **Assembly line and manufacturing (n=290)** | | |
|---|---|---|---|---|---|---|---|---|---|---|
| Block | Variables | Coef. | SE | p-value | Coef. | SE | p-value | Coef. | SE | p-value |
| Socio-demographics (I) | Gender (vs. male) | | | | | | | | | |
| | female | 0.18 | 0.05 | 0.001 ** | 0.06 | 0.03 | 0.041 * | 0.04 | 0.07 | 0.558 |
| | Age (vs. 18-29 years) | | | | | | | | | |
| | 30-59 years | - 0.00 | 0.08 | 0.980 | - 0.01 | 0.04 | 0.740 | 0.04 | 0.09 | 0.724 |
| | 60-67 years | 0.02 | 0.12 | 0.882 | 0.09 | 0.07 | 0.143 | 0.21 | 0.09 | 0.279 |
| General workplace characteristics (II) | Shift work (vs. no) | | | | | | | | | |
| | Yes | - | - | - | - | - | - | - 0.03 | 0.07 | 0.668 |
| Perception regarding the pandemic-related impact in the workplace (III) | Reactance | - 0.07 | 0.02 | <0.001 *** | - 0.07 | 0.01 | <0.001 *** | - 0.06 | 0.02 | 0.002 ** |
| | Information provided by employer | 0.06 | 0.03 | 0.027 * | 0.08 | 0.02 | <0.001 *** | 0.08 | 0.04 | 0.021 * |
| | Trust in colleagues to adhere to distance rules | 0.06 | 0.02 | 0.002 ** | 0.07 | 0.01 | <0.001 *** | 0.09 | 0.02 | <0.001 *** |
| Employee's attitude toward COVID-19 in general (IV) | COVID-19-specific resilience | 0.12 | 0.03 | <0.001 *** | 0.11 | 0.01 | <0.001 *** | 0.09 | 0.03 | 0.002 ** |
| | Affective risk perception | 0.07 | 0.02 | 0.002 ** | 0.11 | 0.01 | <0.001 *** | 0.11 | 0.02 | <0.001 *** |
| Log-like and DF | | - 196.1982 on 644 degrees | | | - 830.2528 on 2578 degrees | | | - 219.3993 on 560 degrees | | |

*p <0.05, **p <0.01, ***p <0.001

All models were controlled for social desirability, 7-day-incidence, company site, and educational background.
No Hauck-Donner effect was found.

***Supplementary File S4:*** Attitude toward organisational, respectively personal, occupational infection control measures

**Attitude toward organisational occupational infection control measures**

| **Professional activity** | | **Office remote (n=336)** | | | **Office on-site (n=1347)** | | | **Assembly line and manufacturing (n=311)** | | |
|---|---|---|---|---|---|---|---|---|---|---|
| Block | Variables | Coef. | SE | p-value | Coef. | SE | p-value | Coef. | SE | p-value |
| Socio-demographics (I) | Gender (vs. male) | | | | | | | | | |
| | female | 0.27 | 0.08 | <.001 *** | 0.12 | 0.04 | .006 ** | - 0.11 | 0.12 | .345 |
| | Age (vs. 18-29 years) | | | | | | | | | |
| | 30-59 years | 0.14 | 0.12 | .244 | 0.06 | 0.06 | .282 | 0.04 | 0.15 | .809 |
| | 60-67 years | 0.16 | 0.17 | .352 | 0.21 | 0.10 | .033 * | 0.65 | 0.28 | .021 * |
| General workplace characteristics (II) | Shift work (vs. no) | | | | | | | | | |
| | Yes | - | - | - | - | - | - | - 0.18 | 0.11 | .093 |
| Perception regarding the pandemic-related impact in the workplace (III) | Reactance | - 0.11 | 0.02 | <.001 *** | - 0.10 | 0.01 | <.001 *** | - 0.10 | 0.03 | .002 ** |
| | Information provided by employer | 0.03 | 0.04 | .537 | 0.06 | 0.02 | .013 * | 0.04 | 0.06 | .481 |
| | Trust in colleagues to adhere to distance rules | 0.02 | 0.03 | .385 | 0.07 | 0.01 | <.001 *** | 0.11 | 0.03 | <.001 *** |
| Employee's attitude toward COVID-19 in general (IV) | COVID-19-specific resilience | 0.09 | 0.04 | .020 * | 0.09 | 0.02 | <.001 *** | 0.13 | 0.05 | .006 ** |
| | Affective risk perception | 0.08 | 0.03 | .013 * | 0.16 | 0.02 | <.001 *** | 0.12 | 0.04 | .001 ** |
| Log-like and DF | | - 305.7062 on 652 degrees | | | - 1372.855 on 2674 degrees | | | - 392.2292 on 602 degrees | | |

*p <.05, **p <.01, ***p <.001

All models were controlled for social desirability, 7-day-incidence, company site, and educational background.
No Hauck-Donner effect was found.

**Attitude toward personal occupational infection control measures**

| **Professional activity** | | **Office remote (n=336)** | | | **Office on-site (n=1347)** | | | **Assembly line and manufacturing (n=311)** | | |
|---|---|---|---|---|---|---|---|---|---|---|
| Block | Variables | Coef. | SE | p-value | Coef. | SE | p-value | Coef. | SE | p-value |
| Socio-demographics (I) | Gender (vs. male) | | | | | | | | | |
| | female | 0.17 | 0.05 | .001 ** | 0.06 | 0.03 | .051 | 0.08 | 0.07 | .252 |
| | Age (vs. 18-29 years) | | | | | | | | | |
| | 30-59 years | - 0.00 | 0.08 | .969 | - 0.02 | 0.04 | .668 | - 0.02 | 0.09 | .787 |
| | 60-67 years | 0.00 | 0.12 | .975 | 0.09 | 0.07 | .181 | 0.19 | 0.17 | .284 |
| General workplace characteristics (II) | Shift work (vs. no) | | | | | | | | | |
| | Yes | - | - | - | - | - | - | - 0.08 | 0.07 | .242 |
| Perception regarding the pandemic-related impact in the workplace (III) | Reactance | - 0.07 | 0.02 | <.001 *** | - 0.07 | 0.01 | <.001 *** | - 0.09 | 0.02 | <.001 *** |
| | Information provided by employer | 0.06 | 0.03 | .024 * | 0.08 | 0.02 | <.001 *** | 0.08 | 0.04 | .024 * |
| | Trust in colleagues to adhere to distance rules | 0.06 | 0.02 | .003 ** | 0.08 | 0.01 | <.001 *** | 0.08 | 0.02 | <.001 *** |
| Employee’s attitude toward COVID-19 in general (IV) | COVID-19-specific resilience | 0.12 | 0.03 | <.001 *** | 0.11 | 0.01 | <.001 *** | 0.07 | 0.03 | .022 * |
| | Affective risk perception | 0.07 | 0.02 | .002 ** | 0.11 | 0.01 | <.001 *** | 0.14 | 0.02 | <.001 *** |
| Log-like and DF | | - 197.1267 on 652 degrees | | | - 865.7844 on 2674 degrees | | | - 253.6176 on 602 degrees | | |

*p <.05, **p <.01, ***p <.001

All models were controlled for social desirability, 7-day-incidence, company site, and educational background.
No Hauck-Donner effect was found.

***Supplementary File S5:*** CONSORT flow-chart

CONSORT
TRANSPARENT REPORTING of TRIALS

## CONSORT Flow Diagram

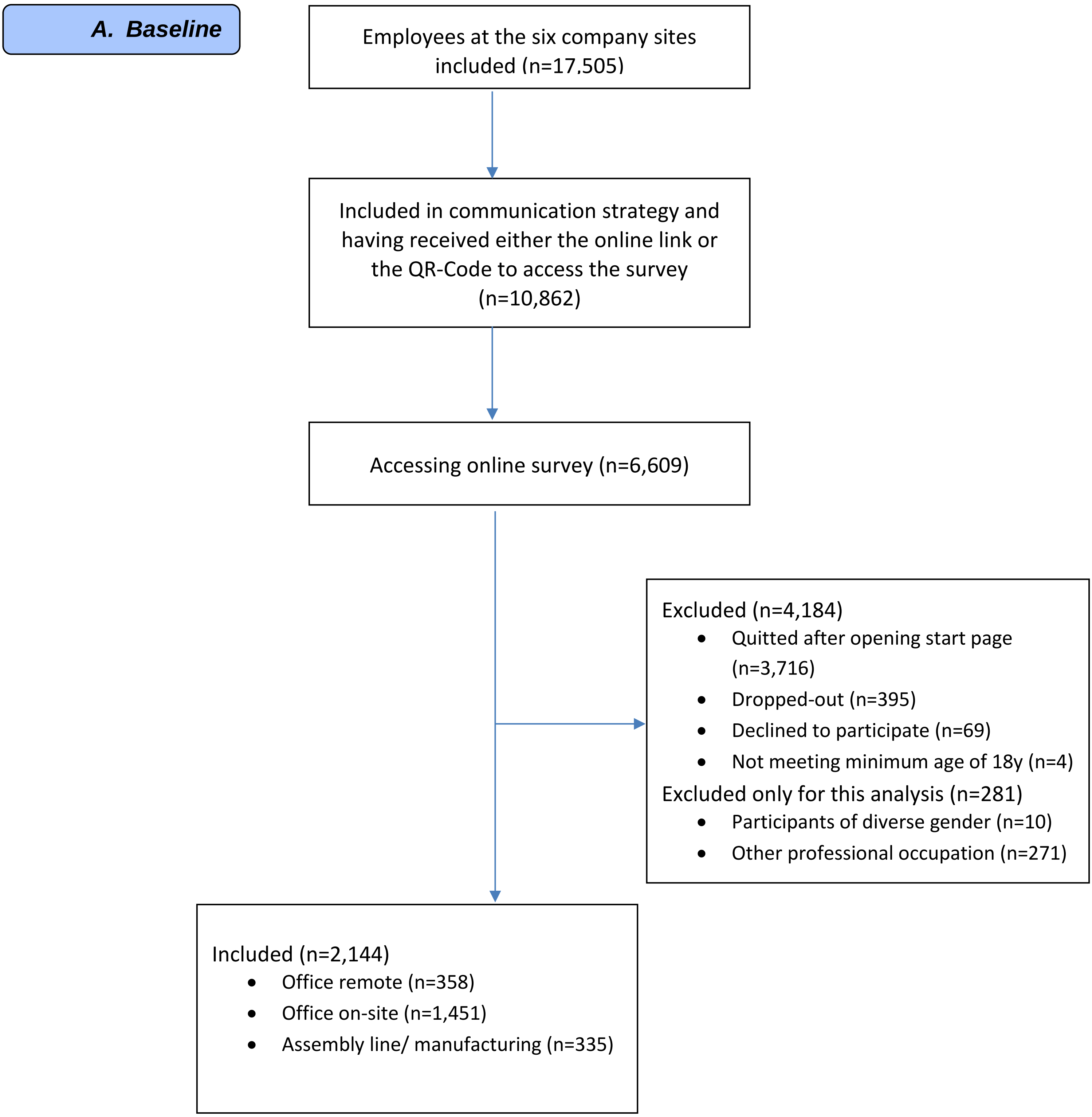

Supplement: Supplementary file 1 [file ijerph-20-00614-s001.zip › ijerph-2072421_SM.pdf]
